# Collateral circulation after revascularization in moyamoya disease: influencing factors and underlying mechanisms

**DOI:** 10.3389/fneur.2026.1795294

**Published:** 2026-07-06

**Authors:** Zhenwei Li, Liming Zhao, Pengpeng Yan, Shangyu Jin, Hao Liang, Ziqiang Liu, Yang Liu, Yuxue Sun, Tao Gao, Chaoyue Li, Gaochao Guo

**Affiliations:** Department of Neurosurgery, Zhengzhou University People’s Hospital, Henan Provincial People’s Hospital, Zhengzhou, China

**Keywords:** angiogenesis, cerebral revascularization, collateral circulation, moyamoya disease, Rnf213

## Abstract

Cerebral revascularization is a primary treatment for moyamoya disease and is generally considered to improve cerebral perfusion while potentially reducing the risk of stroke recurrence. However, the efficiency of postoperative collateral circulation formation varies significantly among individuals, and some patients continue to experience adverse events, such as recurrent ischemia or cerebral hemorrhage, which may be related to inadequate collateralization. Therefore, identifying the factors influencing the formation of collateral circulation after cerebral revascularization is critical for the treatment of moyamoya disease. Previous studies suggest that multiple factors are associated with postoperative collateral vessel formation, including genetic susceptibility (such as the RNF213 p. R4810K variant and microRNA regulation), angiogenic growth factors (such as VEGF, PDGF, and TGF-β1), soluble receptors (e.g., sTie-2), cellular components (including circulating endothelial progenitor cells and mesenchymal stem cells), structural regulators such as Caveolin-1, as well as clinical variables including surgical modality, patient age, and superficial temporal artery blood flow. This article systematically reviews current evidence on factors associated with collateral circulation formation after cerebral revascularization in patients with moyamoya disease, with the aim of informing future research and optimizing treatment strategies.

## 1 Introduction

Moyamoya disease (MMD) is a chronic progressive cerebrovascular disorder characterized by progressive stenosis or occlusion of the terminal portion of the internal carotid artery and the proximal segments of the anterior and middle cerebral arteries, accompanied by the formation of an abnormal vascular network at the base of the skull, which presents a characteristic reticular appearance (“puff of smoke”) on angiography (1). The disease primarily manifests as ischemia and hemorrhage, with pediatric patients predominantly presenting with ischemic symptoms (2), whereas the incidence of hemorrhage is significantly higher in adult patients (3). The pathogenesis of MMD remains incompletely understood; however, immune system dysregulation, inflammation, and genetic factors are considered potential contributors to its development and progression (4, 5). Current evidence suggests that pharmacological treatments have limited effects in delaying or reversing the progression of MMD (6). Cerebral revascularization surgery is the primary therapeutic approach (7, 8). It is thought to improve intracranial blood supply through the external carotid artery system and may reduce hemodynamic stress, thereby potentially lowering the risk of cerebral ischemic and hemorrhagic events.

Cerebral revascularization in MMD can be classified into direct, indirect, and combined approaches. Direct revascularization can immediately increase blood flow to ischemic brain tissue; however, the procedure is technically challenging, and rapid increases in blood flow to chronically ischemic brain tissue may lead to hyperperfusion syndrome with transient neurological deficits (9). In contrast, indirect revascularization is less complex. It provides a gradual, sustained improvement in blood supply to the affected brain tissue, while potentially avoiding ischemia from temporary vessel clamping and prolonged anesthesia, as well as hyperperfusion syndrome resulting from sudden increases in blood flow. However, establishing collateral circulation and improving cerebral blood flow typically require several weeks to months. Evidence suggests that direct revascularization may be associated with improved angiographic vascular reconstruction (10), as well as lower risks of recurrent stroke (11) and long-term hemorrhage (12), compared with indirect revascularization. Combined revascularization integrates the benefits of both direct and indirect methods, and studies have reported more favorable outcomes compared with indirect bypass alone (13). However, clinical observations have revealed a significant variability in the efficiency of collateral neovascularization among patients following indirect or combined revascularization. Bang et al. reported that superficial temporal artery-middle cerebral artery (STA-MCA) anastomosis combined with encephalo-duro-arterio-myo-synangiosis (EDAMS) was associated with a higher extent of revascularization on angiography at 6 months postoperatively, reaching an average extent of 70.8% (14). Kim et al. found that 59.1% of hemispheres demonstrated good neovascularization after encephalo-duro-arterio-synangiosis (EDAS), which was associated with the absence of hypertension and a larger bone flap area (15). Nonetheless, some patients develop poor postoperative neovascularization, resulting in recurrent cerebral ischemia or hemorrhage. Chen et al. reported that the incidence of stroke was higher in patients with poor postoperative collateral circulation formation (PCF) than in those with good PCF, and that may serve as a predictor of long-term prognosis and stroke risk (16).

**Figure 1:**
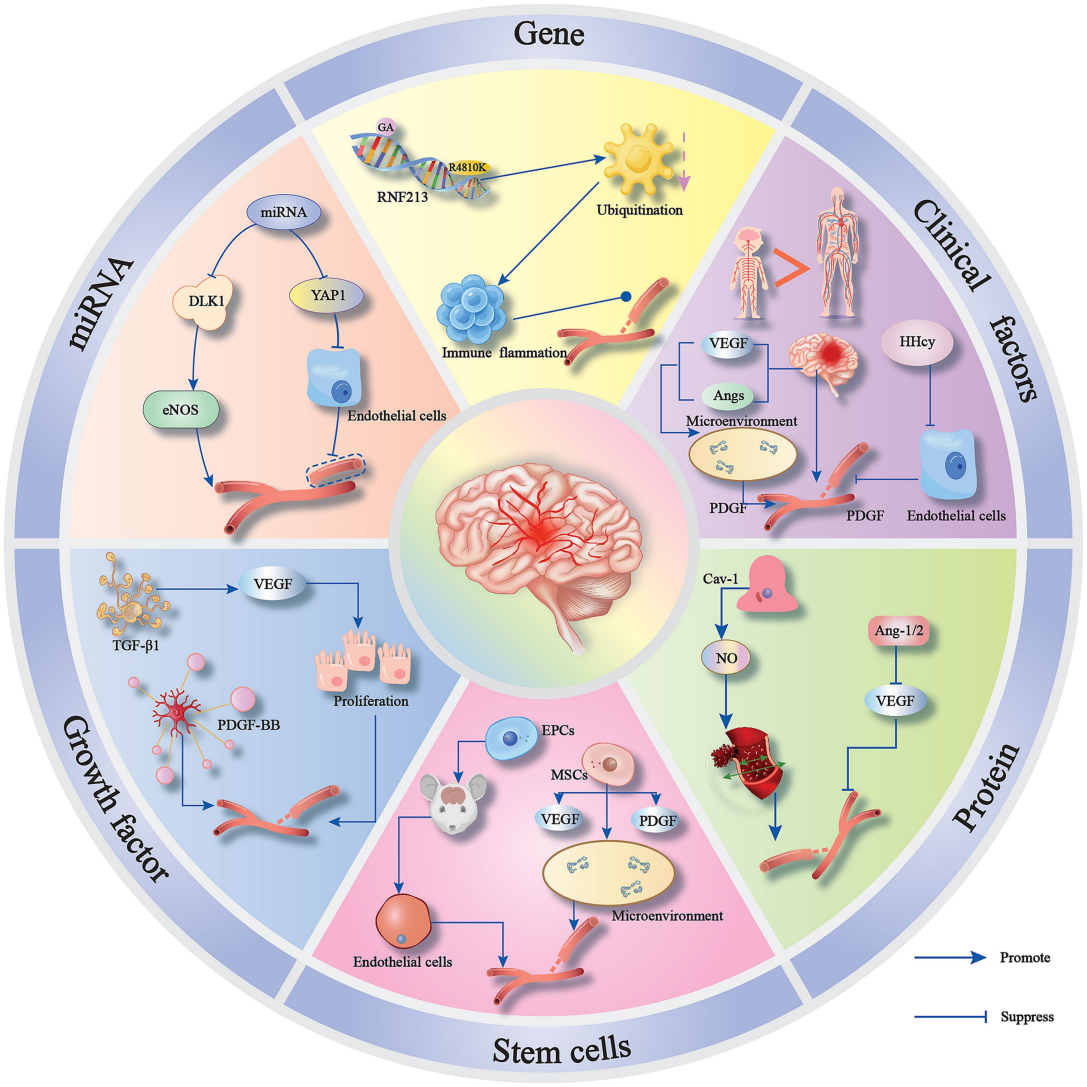
Integrated molecular and cellular mechanisms underlying postoperative collateral circulation formation in moyamoya disease. Postoperative collateral formation (PCF) is a multilevel process shaped by genetic susceptibility conferred by RNF213 variants, putative post-transcriptional regulation by microRNAs, and coordinated angiogenic signaling pathways, including VEGF, PDGF, TGF-β1, and angiopoietin-related signaling. These molecular programs interact with cellular contributors, such as endogenous endothelial progenitor cells and mesenchymal stem cells, as well as the local surgical microenvironment created by cerebral revascularization. Together, these factors modulate endothelial responsiveness and vascular remodeling capacity, thereby influencing the extent of postoperative collateral formation and subsequent clinical outcomes.

Despite increasing research on MMD, the factors influencing collateral vessel generation after cerebral revascularization and the regulatory mechanisms remain incompletely understood. Therefore, this article reviews factors and potential mechanisms associated with collateral circulation formation after cerebral revascularization in MMD (Figure 1).

## 2 Factors associated with collateral circulation formation after revascularization surgery for moyamoya disease

### 2.1 Genetic susceptibility and post-transcriptional regulation

#### 2.1.1 *RNF213* variants and genetic susceptibility

*RNF213* is currently the most extensively studied susceptibility gene for MMD (17, 18) and is located in the q25.3 region of human chromosome 17. Its encoded protein has a complex structure, containing multiple important functional domains, such as the N-terminal stalk, a dynein-like core with six ATPase units, and a multi-domain E3 module (19). Morito et al. reported that the AAA + ATPase module encoded by *RNF213* can dynamically alter its oligomeric state through ATP-binding and hydrolysis cycles, which may contribute to vascular development via intracellular mechanical processes (20). Additionally, the E3 module has been reported to catalyze distinct ubiquitination reactions with the E2 binding enzymes UBE2D2 and UBE2L3. *RNF213* mutations in patients with MMD have been associated with reduced E3 ubiquitin ligase activity and impaired ubiquitination. The most common MMD allele, *RNF213R4810K*, is a dominant negative variant that has been reported to suppress ubiquitination, suggesting that reduced E3 ubiquitin ligase activity may contribute to the pathogenesis of MMD (21). Wu et al. further showed that *RNF213* mutations are associated with MMD susceptibility in a Chinese Han population, with the *R4810K* variant significantly associated with ischemic MMD (22).

Regarding immune-inflammatory responses, the *RNF213*-encoded protein has been reported to interact with infections, immune cells, and cytokines, and may be involved in immune-inflammatory processes, which could be related to the onset and progression of MMD (23–25). The *p. R4810K* variant is closely associated with disease development, and Mineharu et al. identified it as an independent risk factor for contralateral progression in unilateral MMD (26). Furthermore, in a retrospective analysis of adult patients with MMD, Ito et al. found that the *p. R4810K* polymorphism is significantly associated with collateral circulation formation after indirect surgery (27). Ge et al. analyzed 254 patients with MMD and found that those with the heterozygous *p. R4810K* variant (GA) had better PCF than those with the wild-type (GG). Both univariate and multivariate logistic regression analyses showed that GA is associated with good PCF, whereas hemorrhage is linked to poor PCF (28). These findings provide important insights into the factors associated with neovascularization after MMD surgery. From a transcriptomic perspective, future research may further explore the molecular mechanisms by which the p. R4810K variant is associated with neovascularization, as well as its potential regulatory effects on the transcription of related genes. Taken together, current evidence supports an association between RNF213 variants and collateral formation, however, the underlying mechanisms and causal relationships remain to be fully clarified.

#### 2.1.2 MicroRNAs in angiogenesis and collateral formation

MicroRNAs (miRNAs) are a class of small non-coding RNAs, approximately 20–24 nucleotides in length, widely present in eukaryotes. In humans and other animals, miRNA expression is typically tissue-specific and developmental stage-specific. miRNAs primarily regulate gene expression by binding to the 3′ untranslated region of target mRNAs, promoting their degradation or inhibiting their translation (29–31). Changes in miRNA levels are associated with various diseases, including diabetes, obesity, cardiovascular diseases, cancer, and neurodegenerative disorders (32–34). Extracellular miRNAs can be transferred between cells via extracellular vesicles or protein complexes and may regulate gene expression in recipient cells, thereby contributing to physiological and pathological processes such as angiogenesis (32). As a class of non-coding RNAs, miRNAs have been widely implicated in angiogenesis and are reported to be involved in processes such as the proliferation, differentiation, apoptosis, migration, and lumen formation of angiogenesis-related cells (29). They modulate angiogenesis directly by affecting endothelial cell activity or indirectly by regulating the expression of angiogenesis-related proteins (35–38). Huang et al. confirmed that several plasma-secreted miRNAs may serve as biomarkers for MMD (39). miRNAs have also been implicated in collateral circulation formation after MMD surgery. Wang et al. analyzed cerebrospinal fluid from patients with MMD who underwent indirect bypass surgery and found significantly elevated levels of miR-92a-3p, miR-486-3p, miR-25-3p, and miR-155-5p in the angiogenesis group (40). Wen et al. (41) reported that miR-6760-5p is upregulated in the cerebrospinal fluid of patients with MMD and that its overexpression was associated with reduced proliferation, migration, and lumen formation of human umbilical vein endothelial cells (HUVECs). *In vitro* experiments further suggested that upregulation of miR-6760-5p was associated with decreased HUVEC proliferation, migration, and lumen formation, whereas its inhibition was associated with enhanced lumen formation. Western blot analyses showed that miR-6760-5p mimics were associated with decreased YAP1 expression, whereas miR-6760-5p inhibitors were associated with increased expression, suggesting YAP1 as a potential target gene of miR-6760-5p. Further experiments indicated that overexpression of miR-6760-5p combined with transfection of YAP1 overexpression plasmids was associated with enhanced lumen formation and cell migration compared with miR-6760-5p alone. In contrast, transfection of YAP1 small interfering RNA was associated with reduced or abolished lumen formation and cell migration compared with the negative control and miR-6760-5p overexpression groups. These findings suggest that the miR-6760-5p/YAP1 axis may be involved in vascular formation in MMD. Yang et al. (42) found that delta-like factor 1 (*DLK1*) is significantly downregulated in the dura mater of patients with MMD compared with that of patients with aneurysm and confirmed *DLK1* as a target gene of miR-126-5p. *In vitro* experiments suggested that DLK1 was associated with inhibition of endothelial cell proliferation, migration, and angiogenesis, showing effects opposite to those of miR-126-5p. This study reveals the negative regulatory role of *DLK1* in angiogenesis after indirect revascularization surgery for MMD, providing important clues for transcriptomic research. Future studies may further investigate the upstream and downstream gene networks involving DLK1 and miR-126-5p interactions and analyze their roles in angiogenesis-related signaling pathways. Such studies may help to clarify key regulatory genes and potential therapeutic targets for neovascularization after combined revascularization surgery for MMD and provide a basis for improving patient prognosis. Overall, miRNAs may represent potential regulators of collateral formation, although current evidence is largely derived from experimental studies and remains associative.

### 2.2 Angiogenesis-related signaling factors

#### 2.2.1 Vascular endothelial growth factor signaling

Vascular endothelial growth factors (VEGFs) are endothelial cell-specific growth factors that regulate both physiological and pathological angiogenesis (43). They regulate angiogenesis, vasculogenesis, and vascular maintenance during embryonic development and adulthood (44). VEGF contains multiple single-nucleotide polymorphisms that influence its expression. It is a key factor associated with angiogenesis in various intracranial lesions (45–48) and has been reported to be associated with endothelial cell proliferation and sprouting, as well as the formation of microvascular-like structures (49). Wang et al. reported significantly higher serum levels of VEGF in patients with MMD than in healthy controls (50), a finding confirmed by He et al. (51). These findings suggest that elevated serum VEGF levels are associated with the development of abnormal vascular networks at the base of the brain. VEGF expression is also increased in the dura mater of patients with MMD (45), supporting a potential association with the formation of “moyamoya-like” vessels and cerebral collateral circulation formation (50).

Among the VEGF family members, VEGF-A is considered a major pro-angiogenic factor (44), whereas VEGF-B primarily functions as a survival factor and may promote interactions between endothelial cells and pericytes, contributing to the formation of stable cerebral microvessels in damaged regions (52). Park et al. found that patients with the VEGF 2634 CC genotype exhibit better postoperative collateral vessel formation, indicating that VEGF polymorphisms influence collateral vessel formation induced by vascular fusion in MMD and after bypass surgery (53). He et al. (54) identified homocysteine (Hcy) and creatinine as independent risk factors for poor postoperative angiogenesis, and hyperhomocysteinemia is significantly associated with impaired angiogenesis. *In vitro* experiments suggested that Hcy was associated with reduced proliferation, migration, and tube formation of human brain microvascular endothelial cells. In contrast, VEGF could reverse this inhibition, suggesting that VEGF may represent a potential therapeutic target for promoting postoperative angiogenesis. He et al. found that patients with good collateral circulation formation after indirect bypass surgery had lower levels of soluble VEGF receptor-1 (sVEGFR-1) and sVEGFR-2 before and on postoperative day 7, implicating these receptors in collateral circulation after indirect bypass surgery (51). A clinical study by Yang et al. showed that remote ischemic conditioning (RIC) was associated with a reduced incidence of major neurological complications during the perioperative period and increased serum VEGF levels on postoperative day 7, suggesting that RIC may modulate the postoperative angiogenic microenvironment by influencing angiogenesis-related factors (55).

#### 2.2.2 Platelet-derived growth factor pathway

Platelet-derived growth factors (PDGFs) have been implicated in connective tissue remodeling (56). PDGF activates several signaling pathways, including Ras-MAPK, PI3K, and PLC-*γ*, through its receptor tyrosine kinases (PDGFR-*α* and PDGFR-*β*), which are involved in cell proliferation, differentiation, migration, and survival (57). PDGF plays important roles in the development and progression of various vascular and fibrotic diseases (58–63). Aoyagi et al. found that arterial smooth muscle cells of patients with MMD exhibited reduced PDGF receptor expression and a weakened proliferative response to PDGF (64). By inactivating the PDGFR-*α* gene and constructing a mouse model of chronic cerebral ischemia, Hayashi et al. found that PDGFR-α signaling may be involved in spontaneous angiogenesis between the temporal muscle and neocortex after encephalomyosynangiosis (EMS) surgery in MMD (65). In an animal experiment conducted by Marushima et al., VEGF was associated with endothelial cell proliferation, whereas PDGF-BB contributed to vascular stabilization through pericyte recruitment. Balanced co-expression of both factors was suggested to be associated with reduced vascular maturation abnormalities compared with VEGF alone. Co-implantation of PDGF-BB and VEGF into the temporal muscle of a mouse model of chronic cerebral ischemia was associated with enhanced leptomeningeal collateral formation, improved hemodynamics, and increased ischemic tolerance. These findings suggest that EMS surgery combined with myoblast-mediated co-delivery of VEGF/PDGF-BB may represent a potential strategy for enhancing collateral blood flow in chronically hypoperfused brains (66).

#### 2.2.3 Transforming growth factor-β1 signaling

Transforming growth factor-β1 (TGF-β1) is a multifunctional polypeptide growth factor that regulates cell growth and differentiation. It is also a major regulator of connective tissue gene expression and has been implicated in angiogenesis (67, 68). TGF-β1 is highly associated with angiogenesis (68–70). Hojo et al. found that serum TGF-β1 levels were significantly higher in patients with MMD than in healthy individuals, with abundant postoperative neovascularization observed between the temporal muscle and cerebral cortex in all patients, suggesting that TGF-β1 may be involved in the formation of neovascularization in MMD (71). Changshui also found that TGF-β1 expression was significantly higher in patients with MMD than in healthy individuals, indicating its impact on the formation of moyamoya vessels (50).

Furthermore, Chen et al. reported that plasma TGF-β1 levels were higher in patients with ischemic MMD than in those with aneurysm and healthy individuals and were associated with dural collateral formation. Serum TGF-β1 levels were higher in the collateral formation group than in the non-collateral formation group, and plasma VEGF levels were positively correlated with TGF-β1. *In vitro* experiments suggested that TGF-β1 may upregulate VEGF expression in HUVECs, which could contribute to angiogenesis in endothelial cells, indicating that TGF-β1 may be involved in collateral formation in ischemic MMD through VEGF-related pathways (72). These findings suggest that angiogenic factors are associated with collateral formation, however, their precise roles and interactions require further investigation.

### 2.3 Angiopoietin/Tie-2 axis

Tie-2 is a receptor tyrosine kinase primarily expressed in endothelial cells and their embryonic precursor cells. It has been widely implicated in angiogenesis during embryogenesis and in postnatal vascular remodeling. The extracellular domain of Tie-2 is proteolytically cleaved to release a 75 kDa soluble Tie-2 (sTie-2) protein, which binds to the corresponding growth factors in circulation and inhibits angiogenesis (73). sTie-2 is associated with the development of cardiovascular diseases (74, 75). Angiopoietin-1 (Ang-1), a Tie-2 agonist, has been reported to promote vascular maturation by inducing intercellular interactions (73). In contrast, Angiopoietin-2 (Ang-2) is generally considered a context-dependent modulator of Tie-2 signaling and has been associated with endothelial destabilization, which may facilitate the effects of pro-angiogenic growth factors (76). Ang-1, Ang-2, and VEGF collectively regulate angiogenesis, thereby influencing vascular plasticity (77). Chen et al. found that Ang-1, Ang-2, Tie-2, and VEGF are associated with the pathogenesis of MMD. Compared with patients with atherosclerotic cerebrovascular disease (ACVD), those with MMD exhibited lower sTie-2 levels in the middle cerebral artery (MCA) and peripheral plasma. Furthermore, patients with good collateral formation at 6 months postoperatively had lower sTie-2 levels in the MCA and peripheral plasma, suggesting that peripheral plasma sTie-2 levels may have potential value as a biomarker for distinguishing MMD from ACVD and for predicting PCF (78).

### 2.4 Cellular components associated with neovascularization

#### 2.4.1 Circulating endothelial progenitor cells

Circulating endothelial progenitor cells (EPCs) are isolated from human peripheral blood using magnetic bead sorting technology based on cell surface antigen expression and have been proposed to be associated with collateral growth in ischemic tissues (therapeutic angiogenesis) (79). *In vivo* experiments by Iwaguro et al. demonstrated that genetically modified EPCs were associated with enhanced neovascularization in an animal model of limb ischemia (80). Asahara et al. found that even in the absence of angiogenic growth factors, cultured and expanded EPCs effectively promote neovascularization in ischemic tissues (81). Gorla et al. reported that pediatric patients with MMD exhibit elevated levels of circulating EPCs in the peripheral blood and upregulated expression of Ang-2 and VEGF-A in the cerebrospinal fluid, correlating with moderate collateral vascular network development (Suzuki grades III–IV) (82). In a prospective clinical trial of 116 patients with MMD (83), Wang et al. found that EPC count in the peripheral blood was significantly correlated with good PCF, indicating that EPCs may be associated with neovascularization after indirect revascularization surgery (EDAS) for MMD. Wang et al. established a chronic cerebral ischemia model using bilateral internal carotid artery ligation and performed indirect revascularization with encephalomyosynangiosis (EMS). They reported that EMS combined with EPC transplantation was associated with improved cerebral perfusion in rats with chronic cerebral ischemia. This combined treatment was also associated with increased microvascular density, regional blood flow, and brain tissue oxygen partial pressure, along with improved motor function and reduced neuronal damage (84). The findings could provide a theoretical basis for optimizing treatment strategies for MMD. Subsequent transcriptomic studies may help to clarify changes in the expression of neovascularization-related genes under the combined effects of EPCs and indirect revascularization, identify key regulatory genes and signaling pathways, and further elucidate the molecular mechanisms involved.

#### 2.4.2 Mesenchymal stem cells

Mesenchymal stem cells (MSCs) are multipotent cells present in the adult bone marrow with self-replication ability and can differentiate into bone, cartilage, fat, tendon, muscle, and bone marrow stroma (85). MSCs have been reported to be involved in tissue repair in vascular and cardiac diseases by secreting angiogenic, mitogenic, anti-apoptotic, anti-inflammatory, and antioxidant factors (86). They have been investigated in the treatment of various conditions, particularly ischemic diseases such as ischemic cerebrovascular disease and ischemic cardiomyopathy (87–89). Zhang et al. further reported that local MSC transplantation combined with EMS surgery was associated with angiogenesis and improved cognitive function in mice with chronic cerebral ischemia, suggesting that MSC-based approaches may represent a potential strategy for enhancing the efficacy of cerebral revascularization surgery in MMD (90). While cellular components have been implicated in neovascularization, most evidence is derived from experimental models, and their relevance to clinical outcomes remains to be established.

### 2.5 Structural regulators: Caveolin-1

Caveolae are 50–100 nm diameter invaginations of the cell membrane, and are rich in cholesterol and sphingolipids (91). They are abundant in endothelial cells and are involved in endothelial cell transcytosis, vascular permeability, vasomotor tone control, and vascular reactivity (92). As a signature structural protein of the caveolae, Caveolin-1 (Cav-1) has been implicated in tumor growth and ischemic processes through its involvement in embryonic vascular development, normal tissue growth, cell signal transduction, and molecular transport (93, 94). Cav-1 has been reported to interact with endothelial nitric oxide synthase and may modulate nitric oxide signaling, thereby influencing vascular function (95, 96). Gao et al. reported that Cav-1 was associated with reduced cerebral infarction volume, enhanced angiogenesis and neurogenesis, and improved neurological recovery, potentially through the Cav-1/VEGF signaling pathway (97). Zhao et al. found that serum Cav-1 levels in patients with MMD were intermediate between those of stroke patients and healthy controls and increased after combined revascularization surgery. Patients with good PCF had a higher postoperative-to-preoperative Cav-1 ratio, which was positively correlated with cerebral blood flow. *In vitro* experiments suggested that Cav-1 overexpression was associated with enhanced migration and lumen formation of human microvascular endothelial cells, suggesting that Cav-1 may be involved in neovascularization and collateral circulation establishment after combined revascularization surgery and may have potential value as a biomarker for predicting PCF in patients with MMD (98).

### 2.6 Other factors

Surgical revascularization is the primary treatment for patients with symptomatic MMD (6–8) and includes direct, indirect, and combined revascularization. Direct revascularization immediately increases blood flow in ischemic brain regions and rapidly improves hemodynamic status. Indirect revascularization gradually improves blood supply to ischemic brain regions, rationally redistributes blood supply based on the degree of ischemia (6, 99). No consensus currently exists regarding the optimal surgical method for promoting PCF in patients with MMD. Gupta et al. reported that both combined surgery (STA-MCA bypass and EDAMS) and indirect surgery (EDAMS) promote the regression of moyamoya vessels and induce surgical neovascularization, with combined surgery showing superior collateral development (100). Li et al. established an evaluation system based on digital subtraction angiography through a retrospective analysis of 456 patients with MMD to assess collateral vessel generation in the external carotid artery after cerebrovascular revascularization. The study found no significant difference in the amount of collateral vessel generation between direct and indirect cerebrovascular revascularization. However, the level of vessel generation was higher in children than in adults (101).

Additionally, age is an important factor affecting PCF in patients with MMD (15, 54, 83, 102, 103). Chen et al. further found that superficial temporal artery (STA) ultrasound findings correlate with the degree of neovascularization, with STA blood flow exceeding 69.5 mL/min 3 months after bypass surgery, indicating poor collateral circulation compensation (104). Li et al. found that the volume of bloody fluid between the temporal muscle and the target brain cortex after surgery and a history of ischemia are closely related to the establishment of indirect collateral circulation. A large volume of bloody fluid and prior ischemia are potential factors for the postoperative formation of good indirect collateral circulation. These bloody fluids may affect angiogenesis-related cytokines (such as VEGF and angiopoietin) or reflect the donor muscle condition. An appropriate amount of bloody fluid may provide a beneficial microenvironment for collateral circulation (105).

## 3 Discussion

Collateral circulation formation after cerebral revascularization in moyamoya disease is a complex and multifactorial process involving interactions across genetic, molecular, cellular, and clinical levels. This review summarizes current evidence on factors associated with collateral circulation formation after cerebral revascularization in moyamoya disease (MMD), encompassing genetic susceptibility, post-transcriptional regulation, angiogenic signaling pathways, and cellular components. Overall, these findings suggest that collateral formation is a complex, multi-level process involving interactions across molecular, cellular, and clinical domains. At the genetic level, variants in RNF213, particularly p. R4810K, have been consistently associated with disease susceptibility and variability in postoperative collateral formation. At the post-transcriptional level, microRNAs have been implicated in angiogenesis-related processes, although most available evidence is derived from experimental studies. In addition, angiogenic growth factors, including VEGF, PDGF, and TGF-β1, as well as signaling pathways such as the Ang/Tie-2 axis, have been widely associated with vascular remodeling and neovascularization. At the cellular level, circulating endothelial progenitor cells, mesenchymal stem cells, and structural regulators such as Caveolin-1 have also been reported to be involved in angiogenesis-related processes. However, these findings are derived from heterogeneous study designs, and their interactions remain incompletely understood. From a clinical perspective, several biomarkers, including VEGF, sTie-2, EPC counts, and Cav-1 levels, have shown potential associations with postoperative collateral formation. However, the predictive value of these markers remains uncertain, and their clinical applicability requires further validation in large-scale prospective studies.

Although this review primarily focuses on idiopathic moyamoya disease (MMD), the potential relevance of these mechanisms to moyamoya syndrome (MMS), particularly genetically associated forms, warrants further discussion. MMS comprises a heterogeneous group of disorders associated with conditions such as neurofibromatosis type 1 (NF1), ACTA2-related vasculopathy, GUCY1A3 deficiency, BRCC3-related disease, Down syndrome, autoimmune disorders, and prior cranial irradiation. Emerging evidence suggests that several genes implicated in MMS may converge on biological pathways related to vascular remodeling, endothelial dysfunction, smooth muscle cell abnormalities, inflammatory signaling, and angiogenesis, which partially overlap with the mechanistic framework discussed in MMD. For example, NF1-associated MMS has been linked to dysregulated Ras/MAPK signaling and abnormal vascular cell proliferation, whereas ACTA2 mutations are associated with impaired vascular smooth muscle contractility and arterial remodeling. GUCY1A3 deficiency has been implicated in nitric oxide signaling dysfunction and altered vascular homeostasis, while BRCC3-related vasculopathy may involve abnormalities in DNA damage response and endothelial function. These findings suggest that genetically associated MMS may share certain angiogenesis-related and vascular remodeling pathways with idiopathic MMD, although the initiating mechanisms and downstream responses may differ substantially among disease subtypes. At present, however, evidence regarding postoperative collateral formation in MMS remains limited, and most available studies focus predominantly on idiopathic MMD. Therefore, the generalizability of biomarkers and mechanistic findings identified in MMD to MMS remains uncertain. Future studies should further investigate genotype-specific mechanisms of neovascularization and collateral remodeling in MMS, evaluate potential differences in angiogenic responses after revascularization, and determine whether distinct biomarker profiles may contribute to individualized risk stratification and therapeutic decision-making.

This review has several limitations. First, most of the included studies are observational or based on experimental models, limiting the ability to infer causal relationships. Second, the heterogeneity in study populations, surgical techniques, and outcome measures may affect the comparability of findings. Third, many proposed mechanisms are derived from *in vitro* or animal studies, and their relevance to human MMD remains to be fully established. In addition, potential differences between pediatric and adult populations were not systematically addressed.

From a translational perspective, several research directions may have potential clinical relevance. Biomarker-based models integrating genetic, molecular, and cellular factors may help identify patients at risk of poor collateral formation. In addition, angiogenesis-related pathways and cell-based approaches have been explored in preclinical studies and may represent potential therapeutic strategies, although clinical applicability remains uncertain. Future clinical studies should focus on prospective validation of candidate biomarkers using standardized imaging and outcome measures. Early-phase clinical trials may evaluate the safety and feasibility of interventions targeting angiogenesis or cellular therapies. In the long term, randomized controlled trials may be needed to assess their impact on clinical outcomes. Importantly, stratification by disease subtype (MMD vs. MMS), age, and surgical modality should be incorporated into study design.

In summary, current evidence supports an association between multiple biological factors and collateral circulation formation after cerebral revascularization in MMD. However, the underlying mechanisms remain incompletely understood, and further studies are required to translate these findings into clinical applications.
